# Gut transcriptomic changes during hibernation in the greater horseshoe bat (*Rhinolophus ferrumequinum*)

**DOI:** 10.1186/s12983-020-00366-w

**Published:** 2020-07-17

**Authors:** Haijian Sun, Jiaying Wang, Yutong Xing, Yi-Hsuan Pan, Xiuguang Mao

**Affiliations:** 1 School of Ecological and Environmental Sciences, East China Normal University, Shanghai, 200062 China; 2 Institute of Estuarine and Coastal Research, East China Normal University, Shanghai, 200062 China; 3 Key Laboratory of Brain Functional Genomics of Ministry of Education, School of Life Science, East China Normal University, Shanghai, 200062 China; 4 Institute of Eco-Chongming (IEC), East China Normal University, Shanghai, 200062 China

**Keywords:** RNA-Seq, Food intake, Immune system, Intestine, Mammals

## Abstract

**Background:**

The gut is the major organ for nutrient absorption and immune response in the body of animals. Although effects of fasting on the gut functions have been extensively studied in model animals (e.g. mice), little is known about the response of the gut to fasting in a natural condition (e.g. hibernation). During hibernation, animals endure the long term of fasting and hypothermia.

**Results:**

Here we generated the first gut transcriptome in a wild hibernating bat (*Rhinolophus ferrumequinum*). We identified 1614 differentially expressed genes (DEGs) during four physiological states (Torpor, Arousal, Winter Active and Summer Active). Gene co-expression network analysis assigns 926 DEGs into six modules associated with Torpor and Arousal. Our results reveal that in response to the stress of luminal nutrient deficiency during hibernation, the gut helps to reduce food intake by overexpressing genes (e.g. *CCK* and *GPR17*) that regulate the sensitivity to insulin and leptin. At the same time, the gut contributes energy supply by overexpressing genes that increase capacity for ketogenesis (*HMGCS2*) and selective autophagy (*TEX264*). Furthermore, we identified separate sets of multiple DEGs upregulated in Torpor and Arousal whose functions are involved in innate immunity.

**Conclusion:**

This is the first gut transcriptome of a hibernating mammal. Our study identified candidate genes associated with regulation of food intake and enhance of innate immunity in the gut during hibernation. By comparing with previous studies, we found that two DEGs (*CPE* and *HSPA8*) were also significantly elevated during torpor in liver and brain of *R. ferrumequinum* and several DEGs (e.g. *TXNIP* and *PDK1/4*) were commonly upregulated during torpor in multiple tissues of different mammals. Our results support that shared expression changes may underlie the hibernation phenotype by most mammals.

## Background

Some mammals have evolved hibernation to cope with limited food availability and high metabolic energy demands in winter. During hibernation, hibernators spend most of their time in a state known as torpor characterized by dramatic reductions in metabolism, body temperature, heart rate, and oxygen consumption [1, 2]. Periods of torpor are interspersed by metabolically active rewarming phase known as interbout arousals [1] which occur in almost all hibernating mammals [3]. Knowledge about functional changes of organs for adaptation to long-term fasting and hypothermia may provide novel insights into human medicine (e.g. organ preservation).

The gut is the major organ for food digestion and nutrient absorption in the body of animals. Comparing with other organs, the gut is affected greatly by food deprivation during hibernation [4]. It has been shown that fasting can cause atrophy of the intestine with reduction in villus height and crypt depth ( e.g. ground squirrels, [5]; bats, [6]). Studies about the effects of food deprivation and internal food stimuli on the gut have identified important genes, proteins, and molecules which modulate food intake (reviewed in [7], see also [8]). However, most of these studies focused on animals under daily food intake fluctuations. Up to now, studies on the effects of a prolonged fast on the gut function have been rarely conducted in a naturally occurring state, such as hibernation.

Another primary gut function is immunity. It is known that the gut is the largest immune organ in the body of animals [9]. It works the first defense to prevent exogenous pathogens from entering the host cells and tissues [10]. A prolonged fast (e.g. 5–8 months for ground squirrels, [11]) and hypothermia during hibernation have dramatic effects on the intestinal immunity [12]. Although previous studies revealed a substantial decline in the innate and adaptive immune function in multiple tissues during hibernation [reviewed in 13], the classical complement pathway was still active in the intestine of thirteen-lined ground squirrels [13]. Also in ground squirrels, a significant increase of intestinal serum albumin (ALB) expression was observed in winter arousal comparing with summer active [14]. ALB has antioxidant and anti-inflammatory properties [15] and may be involved in innate defense [16]. Previous studies have also shown that interbout arousals from torpor can initiate immune response in ground squirrels [17] and can enhance immune responses to a fungal pathogen in bats [18]. However, little is known about the effect of arousals during hibernation on the intestine immune system. Consequently, more comparative studies will be needed to understand the molecular mechanism of how the intestinal immune system responds to hibernation, in particular to the repeated torpor-arousal cycles during hibernation.

Using proteomic analysis, several differentially expressed proteins have been identified to characterize the hibernating phenotype in the intestine of ground squirrels [14]. However, this previous study only focused on differentially expressed proteins between interbout arousal and summer active. So far, little has been known about genes or proteins whose functions are important in the intestine during torpor. In addition, the previous proteomic screen analysis was only limited to a small number of most abundant and soluble proteins [14]. Thus, transcriptome of intestine based on expression of thousands of genes is quite necessary to investigate molecular interactions underlying the hibernation phenotype in this organ. Recently, comparative transcriptomics have been widely used to identify differentially expressed genes between torpid and active mammals from different species and tissues, such as ground squirrels [19–21], bats [18, 22–24], primates [25, 26], marsupials [27], and bears [28, 29]. In this study, we present the first case of comparative transcriptomics on the gut in a hibernating species of bats.

Almost all insectivorous bats inhabiting in northern latitudes use hibernation to survive the winter. Some of them hibernate for up to eight months without food supply and their body temperatures can be reduced to be below 10 °C [30]. The greater horseshoe bat (*Rhinolophus ferrumequinum*) has a wide distribution across Europe, Africa and Asia [31] and it has become a model system in studying hibernation of bats. Up to now, comparative transcriptomic studies have been conducted on the brain and liver between winter torpid and summer active bats of *R. ferrumequinum* [22, 23]. Additionally, a recent study on this species has indicated that physiological changes during hibernation might alter the gut microbiota community [32]. However, little is known about the response of the gut to hibernation in this species. Here we use comparative transcriptomics to investigate functional changes of the gut during hibernation. In particular, we focus on two primary gut functions: food intake regulation and immunity. By examining the expression differences of thousands of genes among bats from winter torpid, arousal and summer active states, we test three hypotheses: 1) In response to the stress of luminal nutrient deficiency during hibernation, genes and/or gene networks related to regulation of food intake would be upregulated in winter torpor relative to summer active; 2) In order to protect the gut from microbial and inflammatory damage, genes involved in innate immune system will be upregulated in winter torpor and arousal of bats; 3) In order to recover from the torpid state quickly, genes associated with cell proliferation (e.g. cell cycle and cell division) would be overexpressed in winter active bats relative to torpid bats. Moreover, we compared our current results from the gut with previous findings in the brain and liver of *R. ferrumequinum* to investigate molecular effects of torpor on different tissues of hibernating animals. We predicted that differentially expressed genes involved in coping with hypothermia stress would be shared by all of the three tissues.

## Methods

### Experimental design and sampling

**Fig. 1:**
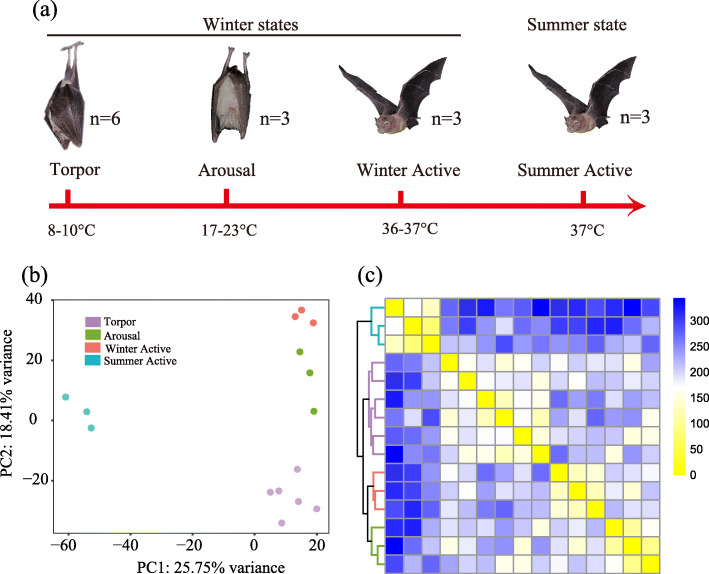
Experimental design and clustering of the 15 *R. ferrumequinum* individuals based on expression data of 14,009 genes. **a** Four physiological states included in this study (Torpor, Arousal, Winter Active, and Summer Active) with their corresponding rectal temperatures; **b** Principal component analysis (PCA) showing gene expression distance of all individuals; **c** Hierarchical clustering analysis based on pairwise Pearson correlation of gene expression in all individuals

In this study we captured 15 adult male individuals of the greater horseshoe bat (*Rhinolophus ferrumequinum*) from Fangshan Cave (39°48′ N, 115°42′ E) in Beijing, China. Three of them were sampled in September when they were active (called Summer Active bats). These active bats were captured when they fly out of the cave just before nightfall. The rest of twelve bats were collected in February when they were torpid with rectal temperatures ranging from 8 °C to 10 °C. Rectal temperature was measured by thermometer (Center 309 data logger). Six of these torpid bats were immediately sacrificed on site (called Torpid bats) when their rectal temperatures were still below 10 °C. To investigate the effects of interbout arousals on animals during hibernation, we imitated the arousal state in the wild by gradually allowing torpid bats to restore their euthermic temperature. Specifically, it took us about two hours to transport the six torpid bats to a hotel near the sampling site. Then we kept these bats in a 27 °C room with air condition and did not supply them any food and water. Three of them were sacrificed in about four hours after capturing when their rectal temperatures were around 20 °C (called Arousal bats). The remaining torpid bats were sacrificed in 24 h after capturing when their rectal temperatures were around 37 °C (called Winter Active bats). Thus, in this study we sampled bats from four different physiological states including three winter states (Torpor, Arousal and Winter Active) and one summer active state (Fig. 1a). It is notable that Arousal and Winter Active here are not truly physiological states occurring in the wild.

For tissue sampling, bats were rapidly sacrificed by cervical dislocation. Then the small intestine near the stomach was taken and transferred to RNase-free PCR tubes. To reduce the amount of gut microbiota included in the host tissue, we collected a small piece of intestine tissue (~ 1 cm). In addition, to reduce the effect of tissue heterogeneity on estimates of gene expression, for each individual we collected two samples from different section of the small intestine. Thus, a total of 30 tissue samples were included in this study (15 individuals × 2 replicates). Tissues were fresh frozen in liquid nitrogen and stored in a − 80 °C freezer.

### RNA extraction, library construction, sequencing and reads trimming

Total RNA was extracted from each of the 30 samples using TRIzol (Life Technologies Corp., Carlsbad, CA, USA) under the manufacturer’s protocol. cDNA library constructions and sequencing on the Illumina Hiseq X ten (paired-end 150 bp) were conducted in Shanghai Majorbio Bio-Pharm Technology Co. Ltd. (four samples) and OE BioTech (26 samples). Raw sequencing reads were trimmed with TRIMMOMATIC version 0.36 [33] using a sliding window of 4 bp with minimum average PHRED quality score of 20 and minimum reads length of 50 bp. A total of 463 Gb clean data were obtained from all 30 samples with an average of 48.9 million clean reads per sample (Table S1). All filtered reads have been deposited into the NCBI Sequence Read Archive database (SRA) (BioProject ID: PRJNA638701).

### Reference transcriptome assembly

We used multiple procedures to obtain a high quality reference transcriptome. First, we generated five assemblies based on 12 libraries from Torpid bats, six from bats in each of the other three states (Arousal, Winter Active and Summer Active), and the combination of all 30 libraries, respectively, using Trinity version 2.8.4 [34] with default settings. For each assembly, redundancy was removed using CD-HIT-EST [35] with 95% sequence similarity threshold and the longest open reading frames (ORFs) were extracted using TransDecoder-5.0.0 (http://transdecoder.github.io). Second, transcripts from each of the five assemblies were combined and redundancy was removed again. Third, the resulting assembly was functionally annotated by searching against the UniProtKB/Swiss-Prot and Pfam database (accessed 16th February 2019) using BLASTx and BLASTp with an E-value cutoff of 1.0E-6. Annotation was further performed by searching against the NCBI non-redundant protein database (nr) database (accessed 16th February 2019) using Diamond version 0.9.24 [36] with a set E-value of 1.0E-6. To eliminate exogenous transcripts, the best hits from the two databases above were used to assign taxonomy to each transcript based on NCBI’s prot.accession2taxid and taxdump mapping file (ftp://ftp.ncbi.nlm.nih.gov/pub/taxonomy/) using R package *taxonomizr*. Gene Ontology (GO) terms for each transcript were retrieved and used for downstream functional enrichment analysis. Only transcripts both functionally annotated and assigned to mammals were included in the final reference transcriptome which comprises of 14,009 genes (22,847 transcripts) with 96.4% of them (13,502) assigned with a GO term (Table S2). Completeness of this reference transcriptome was assessed with BUSCO (Benchmarking Universal Single-Copy Orthologs, [37]) by searching against 4104 conserved orthologous genes from 50 mammalian species. A total of 91.10% BUSCOs were detected in the final reference transcriptome (Table S2).

### Differential expression analysis

Clean reads from each sample were aligned to the reference transcriptome using BOWTIE2 [38] and abundance of transcripts was estimated using RSEM [39] with default settings. Raw read counts across all samples were combined into an expression matrix. Because all samples were sequenced on two different lanes, sequencing batch effect was checked and adjusted using the R package *SVA* [40]. Six surrogate variables were evaluated by comparing a full model matrix (~ four stages) with a null model matrix (~ intercept term). Prior to differential expression (DE) analysis, we filtered those low expressed transcripts with the average read depth < 10. Then we used principal component analysis (PCA) and hierarchical clustering analysis to visualize gene expression variance across all 30 samples based on SVA-adjusted matrix. A pre-PCA analysis revealed that samples from each of the four states (Torpor, Arousal, Winter Active, and Summer Active) grouped together (Figure S1). Despite the existence of the tissue heterogeneity across samples (Figure S1), the difference of the two sampling replicates from the same individual was not significant (Student’s t test, all *P* > 0.05), which indicated that variances of gene expression across all samples were mainly caused by different physiological states. Then we combined the aligned bam files of the two samples from the same individual and quantified the transcripts abundance of the specific individual. Using this method, we re-evaluated expression variance across all 15 individuals using PCA and hierarchical clustering analysis as above.

The inclusion of the two non-independent replicates from the same individual during DE analysis may cause the pseudoreplication problem and underestimate the within-group variation of individuals in each physiological state. In order to reduce the type I error rate caused by pseudoreplication problem [41], we account for the correlation of variation within individual using a randomized blocking design. Specifically, we randomly assign the two samples from the same individual to “PartA” and “PartB” and incorporate the blocking design into differential expression tools with ~sampled part + physiological states. By fitting design model above, we can compare the four physiological states with adjustment of the baseline differences from tissue heterogeneity.

Two methods, edgeR [42] and DESeq2 [43], were used to identify differentially expressed genes (DEGs). To reduce false positives, a gene was considered to be differentially expressed only when it was significant in both edgeR and DESeq2 with a false discovery rate (FDR) < 0.05 and log_2_ transformed fold change (Log_2_FC) of > 1 or < − 1. To determine the biological role of DEGs identified in each comparison, we performed functional enrichment analysis using the REACTOME, KEGG pathway database and Gene Ontology (GO) [44] in the R/BIOCONDUCTOR package *clusterProfiler* [45]. Significance is determined with the Bonferroni corrected *P* value of < 0.01.

### Weighted gene co-expression network analysis (WGCNA)

We next identified modules (groups of co-expressed genes across samples) that differentiate each of the four sates in an unbiased way using a weighted gene co-expression network analysis (WGCNA [46];). Our WGCNA focused on 1614 DEGs identified in all pairwise comparisons of the four states above (see Results). Specifically, the correlation between genes’ expression was transformed to connection strengths using a power function with a thresholding power of β = 12. The resulted adjacency matrixes were used to calculate topological overlap matrix (TOM) and corresponding dissimilarity matrix (1-TOM). Then a cluster dendrogram was built by a dynamic tree cutting algorithm [47] to identify modules with a minimum 25 genes per module. Similar modules were merged using a module dissimilarity threshold of 0.25. The module eigengene [48] was used to summarize the expression profiler of a given module. Association between modules and traits were quantified by correlate eigengenes (summary profiles for each module) and evaluated by BH adjusted *P*-values (q-value, < 0.01). We calculated the module membership value of a gene (kME, the Pearson correlation between its expression value and the module eigengene) which was used to identify the most important intra-module hub genes associated with phenotypic traits [47, 48]. To determine the biological functions of genes in each module, we performed functional enrichment analysis using the same procedures as above. To further investigate the molecular interactions of genes in each module, we conducted protein-protein interaction (PPI) network analysis with STRING version 11.0 [49] and cytoscape version 3.8.0. (https://cytoscape.org/).

## Results

### Differential expression analysis

**Fig. 2:**
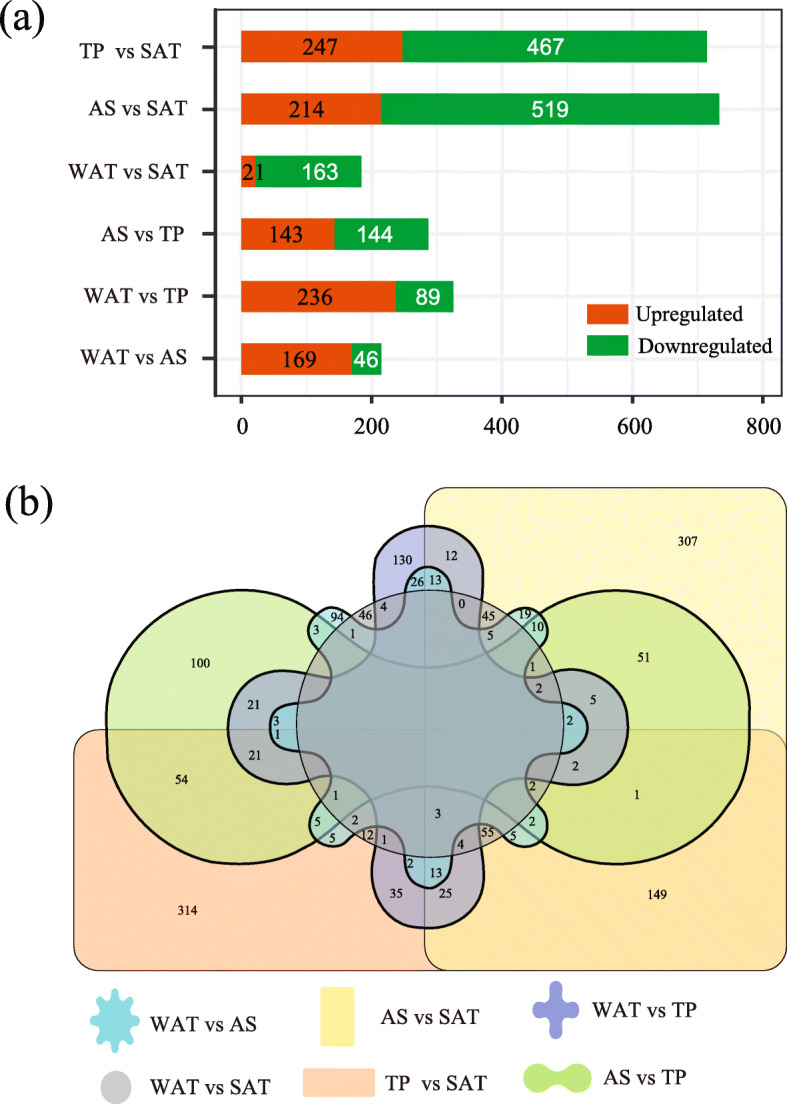
**a** Bar graph showing the number of differentially expressed genes (DEGs) identified in each pairwise comparison of the four states. **b** Venn diagram showing the number of DEGs shared between each pairwise comparison of four physiological states. TP vs SAT: Torpor vs Summer Active; WAT vs SAT: Winter Active vs Summer Active; AS vs SAT: Arousal vs Summer Active; WAT vs TP: Winter Active vs Torpor; AS vs TP: Arousal vs Torpor; WAT vs AS: Winter Active vs Arousal

The principal component (PCA) and clustering analysis both revealed that individuals from each of the four states (Torpor, Arousal, Winter Active, and Summer Active) grouped together respectively (Fig. 1b and c). To account for gene expression variation of the two replicates from the same individual (Figure S1), we included all 30 samples in the following DE analysis. However, to reduce the type I error rate caused by pseudoreplication problem, we used a randomized blocking design when performing DE analysis (see details in Materials and methods). Under this blocking design, EdgeR and DESeq2 identified almost the same number of differentially expressed genes (DEGs) in each comparison (Figure S2, Table S3 and S4). In this study only shared DEGs identified by both methods were considered as true DEGs (Fig. 2a and b). After removing the overlapped DEGs identified in all six comparisons, a total of 1614 DEGs were retained (Fig. 2b, Table S5).

For upregulated DEGs, significant enrichments of GO terms and/or KEGG and Reactome pathways were detected in Torpor, Winter Active and Arousal relative to other states (Figure S3, Table S6 and S7). We found that those DEGs upregulated in Torpor relative to Summer Active were associated with insulin-like Growth Factor (IGF), and those DEGs upregulated in Winter Active relative to Arousal were associated with carbohydrate metabolism (Figure S3 and Table S7). Those DEGs upregulated in Arousal and Winter Active comparing with Torpor or Summer Active were associated with metabolism of lipids, carbohydrate, cofactors, and vitamins, biological oxidations, digestion, apical plasma membrane, and carbohydrate transmembrane transporter activity (Figure S3, Table S6 and S7). For downregulated DEGs, we found significant enrichments of GO terms (Table S6) and/or KEGG and Reactome pathway (Figure S4 and Table S7) in five comparisons. Among those downregulated in Torpor, Arousal and Winter Active relative to Summer Active (*n* = 369), a large proportion of them (230, 62.3%) were associated with immune function (Table S6 and S7). Those downregulated in Arousal and Winter Active relative to Torpor were associated with metabolism of proteins, hemostasis and complement and coagulation cascades (Figure S4, Table S6 and S7).

### WGCNA

**Fig. 3:**
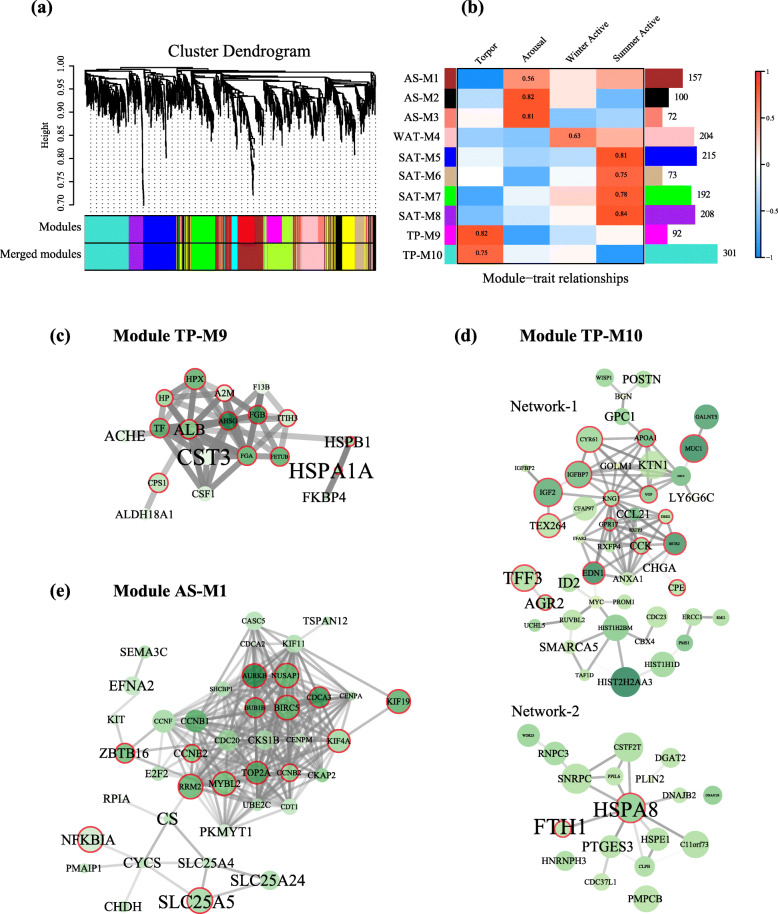
Weighted Gene Coexpression Network Analysis (WGCNA). **a** Cluster dendrogram built based on 1614 DEGs in WGCNA. All modules and merged modules with a module dissimilarity threshold of 0.25 were shown in upper and down panel, respectively. **b** Heatmap showing correlations between module eigengenes and each of the four states. The color scale indicates the strength of correlation. Number of genes included in each module was shown in the bar plot right; **c**-**e** Results of protein-protein interaction (PPI) network analysis on DEGs in three WGCNA modules (TP-M9, TP-M10 and AS-M1 modules; c, d and e). Size of nodes indicates module membership values (MM) of specific DEGs in corresponding WGCNA modules. Size of the label indicates the mean expression level (log_2_CPM) across the samples. Proteins discussed in Results are cycled with red color. Edge transparency indicates the interaction score between two proteins (less transparent stronger correlation of the two proteins)

WGCNA based on 1614 DEGs yielded ten modules with the number of genes ranging from 72 to 301 per module (Fig. 3a, b; Table S5). Among these modules, six were found to be positively associated with three winter states (TP-M9 and TP-M10 modules for Torpor; AS-M1, AS-M2 and AS-M3 modules for Arousal; WAT-M4 module for Winter Active). For Summer Active state, we identified four significantly positive modules (SAT-M5, SAT-M6, SAT-M7, and SAT-M8 modules).

We found significant GO terms and/or pathways in genes from three modules (Figure S5, Table S8). Specifically, genes in TP-M9 and/or TP-M10 module were associated with Insulin−like Growth Factor (IGF), protein activation cascade, and hemostasis, whereas genes in AS-M1 module were enriched in the process of cell cycle and mitosis. We further conducted PPI network analysis on genes from modules associated with the three winter states to investigate molecular interactions of these genes. PPI networks were identified among genes from three modules, which will be discussed in details below.

First, one network including 19 proteins was constructed in TP-M9 module (Fig. 3c and Table S9). Among these proteins, eight of the top 10 proteins with the largest expression difference between Torpor and Summer Active belong to acute-phase proteins (APPs) (AHSG, ALB, FETUB, FGA, FGB, HP, HPX, and TF). Another two are heat shock proteins (HSPA1A and HSPB1). All of these APPs are in the list of the top 10 proteins with the largest WGCNA module membership (MM) value. The rest two, CPS1 and ITIH3, are associated with urea cycle and hemostasis, respectively. In addition, seven APPs are also in the list of the top 10 proteins with the largest degree of connection (K) value in PPI network. The rest three, CST3, CSF1 and A2M, are associate with protective function and hemostasis. Last, seven proteins above (A2M, ALB, CST3, CSF1, HSPA1A, HSPB1, and TF) are also in the list of the top 10 proteins with the highest mean expression value across Torpid samples.

Second, two networks including 66 proteins were generated in TP-M10 module (Fig. 3d and Table S10). For Network-1 (48 proteins), we mainly focused on those proteins in the list of the top 15 with either the largest expression difference between Torpor and Summer Active, the highest mean expression value across Torpid samples, the largest MM value or K value. Thirty-six such proteins are included. Among them, five and two proteins are associated with regulation of food intake (CCK, CPE, DRD2, GPR17, and VGF) and digestion (SSTR2 and APOA1), respectively. All of these seven proteins are centered by KNG1 which shows the largest K value (Fig. 3d, Network-1). TEX264 is also centered by KNG1 and related to ER-phagy. One (CYR61) may be involved in restoring mucosal homeostasis after intestinal injury [50]. Three (MUC1, AGR2 and TFF3) are related to maintenance and repair of the intestinal mucosa. AGR2 and TFF3 also have the largest mean expression value across Torpid samples. It is notable that another intestinal defense protein (MUC2) and a histone protein (HIST2H2AA3) show the largest expression difference between Torpor and Summer Active, and the later histone protein also exhibits the largest MM value. Another one protein (EDN1) has an effect on ischemic stroke [51]. We also found two insulin-like growth factors (IGF2 and IGFBP7). For Network-2, a heat shock protein (HSPA8) and an iron storage protein ferritin (FTH1), have the largest mean expression value across Torpid samples, and TXNIP, another highly expressed proteins (top 5 in the list of 66 proteins above), but not in the network, is a potent antioxidant.

Third, we identified one network in AS-M1 module including 41 proteins (Fig. 3e and Table S11). Among the top 10 upregulated genes in Arousal vs. Torpor, nine are involved in cell cycle and mitosis (AURKB, BIRC5, BUB1B, CDCA3, CCNB1, NUSAP1, RRM2, TOP2A, and ZBTB16). The left one, KIF19 plays an important role in regulation of the length of motile cilia. NFKBIA has the largest MM value and is related to immune function. Among the other top 10 proteins with the largest MM, five were also in the list of the top 10 upregulated genes in Arousal vs. Torpor (AURKB, BIRC5, CCNB1, NUSAP1, and RRM2), and together with another four proteins (CCNF, KIF4A, MYBL2, and SLC25A5), are all involved in cell cycle and mitotic process. The left one, KIF19 was also in the top 10 upregulated genes in Arousal vs. Torpor as described above. Among the above proteins, SLC25A5 has the highest expression value in Arousal. Last, all of the top 10 proteins with the largest K value are associated with cell cycle and mitosis.

## Discussion

To our knowledge, this is the first study to assess effects of hibernation on the gut of animals in natural conditions using comparative transcriptomics. We identified 1614 differentially expressed genes (DEGs) during four physiological states (Torpor, Arousal, Winter Active, and Summer Active). Using a weighted gene co-expression network analysis (WGCNA), we assigned 926 DEGs into six modules associated with the three winter states. Our current results include all of the proteins (ALB, HSPA8, APOA1, and HMGCS2) that showed over two-fold expression changes in winter Arousal vs. summer Active in the intestine of ground squirrel [14]. This consistency supports the reliability of our current results.

In this study we observe overexpression of genes in winter Torpor or Arousal whose functions are related to several important aspects of the gut, such as intestinal defense (*MUC1*, *MUC2*, *AGR2*, and *TFF3*), cilia formation (*KIF19*), responses to ischemia (*EDN1*), and cell cycle and mitosis (see details in Results). In order not to be lost in the map, here we focus on transcriptomic changes of two primary gut functions: regulation of food intake and immune response.

### Regulation of food intake in the gut during torpor

Because the gut is the major organ for nutrient absorption in animals, we predict that the deficiency of luminal nutrient during hibernation may induce overexpression of those genes that reduce food intake or suppress the appetite. Consistent with this prediction, we find that at least six genes in TP-M10 module (*CCK*, *CPE*, *GPR17*, *VGF*, *RXFP4*, and *DRD2*) are involved in regulation of food intake. Proteins encoded by these genes are mostly centered by KNG1 in the protein-protein interaction network (PPI network, Fig. 3d and they were all upregulated at least four-fold in Torpor vs. Active. Below we will discuss their functional roles in food intake regulation one by one.

**Fig. 4:**
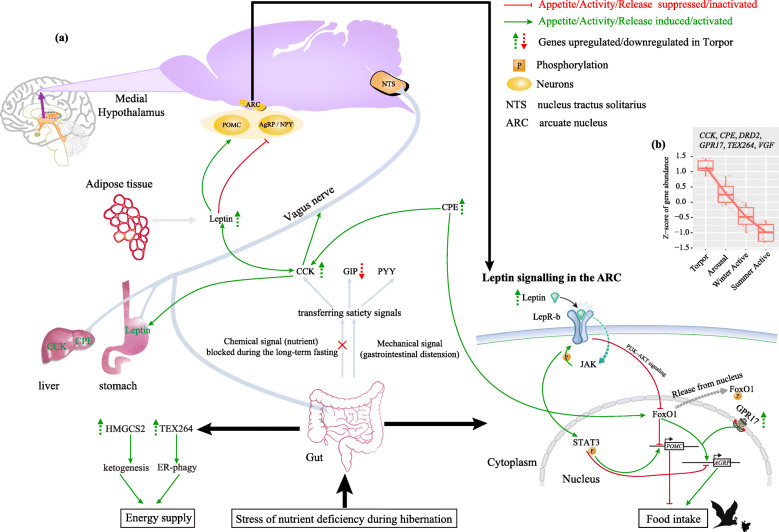
Schematic view of the molecular interactions of genes/proteins involved in response to the stress of luminal nutrient deficiency during hibernation. **a** The molecular interactions of DEGs in energy supply and regulation of food intake by transferring satiety signals between central nerve system and the intestinal afferent vagus nerve, and Leptin signaling in the ARC (arcuate nucleus). **b** Cluster of six DEGs (*CCK, CPE, DRD2, GPR17, TEX264,* and *VGF*) associated with food intake regulation based on expression patterns across the four states

CCK (cholecystokinin) is one of the well-known gut hormones which act as important satiety signals mediator in controlling food intake and regulating absorption of nutrients [52, 53]. It is known that the release of CCK is stimulated by nutrient signals from food digestion [54, 55]. However, these nutrient signals may be blocked during the long-term fasting in hibernating animals. Recently, gastrointestinal (GI) distension, as an afferent signal, has been proved to be more sensitive in regulating food intake than nutrient-stimulated signals in GI tract [56]. Thus, GI distension may serve as a mediator transferring satiety signals and stimulate the release of CCK [56, 57]. Interestingly, *CCK* is the only upregulated gene among the three hormones secreted from the intestine [*CCK*, *GIP* and *PYY*, 59] that induce satiety signals to the nucleus tractus solitarius (NTS) though vagal afferents [58]. Among them, *GIP* was downregulated in Torpor relative to Active, and *PYY* was not differentially expressed in this study. In addition, CCK can modulate the sensitivity of gastric afferents to leptin [59] and inhibit gastric emptying in animals [60]. In rats CCK mediates satiety signals by inducing the release of leptin stored in stomach [61]. Leptin, a steroid hormone, in return, can enhance satiating effect of CCK [57] and the role of leptin in decreasing food intake has been well documented in mammals [8]. Later studies have proposed that there is a synergistic interaction between CCK and leptin to reduce food intake [7, 62]. Consistent with this proposal, we found that *LEP*, encoding leptin, was expressed with the second largest fold change among all upregulated genes in Torpor relative to Active (nearly 20-fold) although it was identified as the DEG only by DESeq2 (Table S4). Consistent with our finding, CCK was also significantly elevated in Torpor vs. Active in the liver of *R. ferrumequinum* ([23], Table S12]). *CPE* (carboxypeptidase E), an obesity susceptibility gene, has been shown to be responsible for the processing of pro-CCK into its bioactive forms [63]. Stimulation of CPE can increase energy efficiency [64] which is essential when energy is limited during hibernation. The importance of CPE for hibernating animals is further supported by the observation that it was upregulated in Torpor vs. Active in brain and liver of *R. ferrumequinum* ([22, 23]; Table S12). GPR17, a G protein-coupled receptor, acts as a target of FoxO1 to regulate appetite and energy expenditure [65]. The expression of *FOXO1* can be induced in response to fasting [66, 67]. However, in the current study *FOXO1* was not differentially expressed in all six comparisons and it maintained a high expression level in each of the four physiological states with a mean log_2_CPM value of 5. STAT3, an antagonism to FoxO1, also maintained a high expression level in all of the four states and showed the highest expression in Torpor (mean log_2_CPM > 7). Leptin can inactivate FoxO1 and reduce expression of *GPR17* in nucleus. The inactivation of FoxO1 allows STAT3 to inhibit the expression of *AGRP* and stimulate the expression of *POMC* [68], which results in increased sensitivity to insulin and leptin and reduced food intake [68]. Additionally, FoxO1 also regulates CPE to integrate food intake with energy expenditure [64]. In summary, our current results suggest that long-term fasting and energy depression during hibernation may first induce the release of endogenous hormones (e.g. CCK and Leptin) which can help to regulate food intake by triggering the circuit between central nerve system and the intestinal afferent vagus nerve. To clearly illustrate the role of genes/proteins discussed above in food intake regulation, we summarized their functional interactions as in Fig. 4a (Modified from [57, 68]).

VGF, a neuronal and endocrine peptide precursor, and its derived peptides can decrease food intake [69–71]. It was notable that this gene was significantly downregulated in Torpor vs. Active in the brain of *R. ferrumequinum* [22]. This may be caused by differential roles of VGF in the gut and brain [72]. Another G protein-coupled receptor, RXFP4, is expressed in the enteric nervous system and its ligand is the gut novel hormone insulin-like peptide 5 (INSL5). A recent study found that fasting and long-term energy deprivation can induce the expression of *INSL5* which enhances appetite by stimulating RXFP4 [73]. It was notable that *INSL5* was almost not expressed in the gut. The last one, DRD2 can also mediate food intake via dopamine signaling [74].

Another way to respond to stress of nutrient deficiency or starvation is to activate endoplasmic reticulum-phagy (ER-phagy). This selective autophagy helps mammalian cell to acquire amino acids and other cellular components by selectively transporting cytosolic contents to lysosomes for degradation. Two recent studies have proved that *TEX264* is a critical determinant of ER-phagy in response to nutrient deprivation ([75, 76]; see also Fig. 4). In addition, fasting induces an overexpression of *HMGCS2* in Torpor (almost 5-fold in Torpor vs. Active, Table S3 and S4). The encoded protein by this gene is a key enzyme regulating ketone body production. The increased ability for ketogenesis can produce adequate energy supply for the gut. Consistent with our result here, HMGCS2 was also elevated 2.2-fold in winter Arousal vs. summer Active in the intestine of ground squirrels [14].

Finally, we found that six DEGs (*CCK, CPE, DRD2, GPR17, TEX264,* and *VGF*) showed a decreased expression level across the four states from Torpor to Summer Active (Fig. 4b), corresponding to an increase of energy demand from food intake along the rise of body temperature. This implies that genes regulating food intake and energy supply may be important in triggering cyclic variation of energy demand during hibernation.

### Regulation of immune system in the gut during hibernation

Our current study revealed a large proportion of downregulated DEGs in three winter states relative to Active were associated with immunity (Table S6 and S7), supporting a systemic suppression of immune function during hibernation [77]. However, consistent with our second hypothesis, we found that several upregulated genes in Torpor or Arousal were related to innate immunity.

First, among the 30 proteins in the PPI network of TP-M9 module (Fig. 3c and Table S9), eight of the top 10 upregulated proteins in Torpor vs. Summer Active belong to acute-phase proteins which play important roles in the early response to infection or tissue injury [78]. Among them, AHSG (also known as FETUA) and FETUB belong to the set of negative acute-phase genes [79] and they play anti-inflammatory roles by counter-regulating the innate immune response [80]. It is notable that AHSG shows the largest expression difference between Torpor and Summer Active and also the largest K value. ALB has the second largest K value and its protein (Albumin) has antioxidant and anti-inflammatory properties [15]. This protein exhibited the greatest fold change in the intestine of ground squirrels in winter Arousal vs. summer Active [14]. Similarly, APOA1 was also overexpressed in hibernating ground squirrel intestine [14] and has potential effects of anti-inflammatory. Hemopexin encoded by *HPX* exhibits the biggest MM value. This protein is a heme-binding plasma glycoprotein and can act as an antioxidant against oxidative damage mediated by hemoglobin [81]. Haptoglobin encoded by *HP* also acts as an antioxidant and anti-inflammatory [82]. Similar to hemopexin, it also plays a crucial role in against hemoglobin-mediated oxidative damage [83]. Another important protein in the network of TP-M9 module is CSF1, macrophage colony-stimulating factor, which can restore innate immunity after injury of liver in mice [84]. Last, KNG1 has the largest K value in the networks of proteins in TP-M10 module. This protein has been proved to be involved in inflammation, coagulation, and innate immunity [85]. Consistent with our findings above, previous studies on the intestine and lung of thirteen-lined ground squirrels showed that certain aspects of innate immunity were still maintained during torpor [13, 86]. Another study on the bone marrow of thirteen-lined ground squirrels revealed a shift to innate immune responses during hibernation [21]. Overall, our current and previous studies support that innate immune system plays an essential role in protection against infection or tissue injury during torpor.

Second, among the proteins in AS-M1 module (Table S5), NFKBIA exhibits the largest MM value and this protein has been reported to alleviate gut infection by reinforcing innate immune response via the NF-kB system [87]. Another protein, CEBPD, is an important transcription factor regulating the expression of genes involved in immune and inflammatory responses. This protein has been shown to play an essential role in Gamma-tocotrienol mediated protection against ionizing radiation (IR)-induced hematopoietic and intestinal injury [88]. A further protein (GGT1) also plays an important role in cell antioxidant defense mechanism [89]. Accordingly, our current results support an important role of interbout arousals from torpor in enhancing immune responses during hibernation [17, 18].

It is notable that *ACE2*, encoded a receptor for human coronaviruses SARS-CoV and 2019-nCoV [90], was downregulated over 2 fold in Torpor relative to Active (Table S4) although currently we have no idea about its effect on the interaction of ACE2 with coronaviruses during hibernation.

### Comparing with previous studies

In order to test whether hibernation has a differential effect on different tissues of a hibernating animal, we compared our current results with previous comparative transcriptomics on the brain and liver in *R. ferrumequinum* [22, 23]. Among upregulated DEGs in Torpor vs. Summer Active, we found two DEGs (*CPE* and *HSPA8*) overlapped by the three tissues. In addition, we identified 11, 13 and 32 DEGs shared by the gut and liver, the gut and brain, and the brain and liver, respectively (Table S12).

Consistent with our prediction that shared DEGs by multiple tissues will be important in coping with hypothermia during hibernation, we found one head shock protein (HSPA8) upregulated in all of the three tissues. This heat shock protein has the second largest expression value in TP-M10 module (Table S10, Fig. 3d) and was also significantly overexpressed in winter Arousal vs. summer Active in the intestine of ground squirrels [14]. These suggest that this protein may belong to ‘protective’ protein during hibernation. In addition, other heat shock proteins were found in the list of shared upregulated genes in torpor vs. active between the gut and liver (HSPB1) or between the brain and liver (HSP90B1, HSP90AA1, HSPA4L, and HSPH1) (Table S12), suggesting their general roles in protection against stress during torpor [12].

By comparing with other previous studies, we found that many of the shared upregulated genes in torpor vs. active by two of the three tissues in *R. ferrumequinum* were also reported to be important during torpor in other hibernating mammals. For example, *TXNIP*, one of the 11 shared upregulated genes between the gut and liver (Table S12), encodes thioredoxin-interacting protein which is a potent antioxidant. This gene has been shown to be significantly elevated in torpor vs. active in all three tissues (liver, brain and muscle) of small South American marsupials [27] and was also over three fold higher in torpor vs. active in the bone marrow of thirteen-lined ground squirrels [21]. Another example is *PDK1*, one of the 13 shared upregulated gene between the gut and brain (Table S12). Its encoded protein, PDK1, is a protein kinase. PDK1 and its other isozymes (PDK 2, 3 and 4) phosphorylate pyruvate dehydrogenase (PDH) and inhibits its activity [91, 92], which promotes the shift from carbohydrate metabolism to lipolysis. Suppression of PDH activity by upregulation of PDK isozymes (PDK 1 and 4) has been well documented in multiple tissues of different hibernating mammals (e.g. white adipose tissue in dwarf lemurs, [26], brain in marsupial, [27], liver and skeletal muscle in ground squirrels, [93]). The commonality of overexpression of these genes in torpor in different tissues and different mammals further supports that genes and biochemical process underlying the hibernation phenotype may be shared by all mammals (see also [94]).

## Conclusions

This study has three major findings. First, we identified candidate genes that are important in response to the stress of luminal nutrient deficiency during hibernation. Our PPI network results suggest that they are interacted with each other in regulation of food intake. Second, we detected overexpression of genes in Torpor and/or Arousal whose functions are involved in innate immune response. Such genes may be important in preservation of organ function during extended hypothermia. Third, by comparing with previous studies, we find several important genes whose expressions are commonly elevated during torpor in multiple tissues of different hibernating mammals. Thus, our findings in this study can be applicable to other hibernating mammals.

It is now known that the gut microbiota plays essential roles not only in food digestion, energy harvest and metabolism, but also in immune function of their hosts [95, 96]. The host-microbial cross-talk is particularly dynamic in hibernating mammals with a prolonged fast [97]. Previous studies on the gut microbiota in hibernating mammals have shown that the structure and composition of the gut microbiota have changed greatly during hibernation (e.g. in ground squirrels, [98], in horseshoe bats, [32]). In the future, functional comparisons of the structure and composition of the gut microbiota between summer active and winter torpid animals will be important if we aim to understand the role of molecular interactions of the gut and microbiota in response to stressful conditions (e.g. extended fasting and hypothermia) during hibernation.

## Supplementary information

**Additional file 1: Figure S1.** Principal component analysis (PCA) and clustering of the 30 samples from 15 individuals based on expression data of 14,009 genes showing gene expression distance of all samples.

**Additional file 2: Figure S2.** Number of differentially expressed genes (DEGs) identified in each pairwise comparison of the four states by DESeq2 and edgeR, respectively.

**Additional file 3: Figure S3**. Results of GO terms and/or KEGG and Reactome pathway enrichment analysis on upregulated DEGs identified in four comparisons. Description of terms or pathways with same color indicates similar function.

**Additional file 4: Figure S4.**Results of KEGG and Reactome pathway enrichment analysis on downregulated DEGs identified in five comparisons. Description of terms or pathways with same color indicates similar function.

**Additional file 5: Figure S5.**Results of GO terms and/or KEGG and Reactome pathway functional enrichment analysis on genes in TP-M9, TP-M10 and AS-M1 modules identified in WGCNA. Description of terms or pathways with same color indicates similar function.

**Additional file 6: Table S1.** Sequencing data collected from RNA-seq. **Table S2.** Summaries of assemblies generated by TRINITY.

**Additional file 7: Table S3.** Details of DEGs identified in each pairwise comparison of four physiological states using edgeR.

**Additional file 8: Table S4.** Details of DEGs identified in each pairwise comparison of four physiological states using DESeq2.

**Additional file 9: Table S5.** Results of WGCNA module detection of 1614 DEGs and mean log_2_CPM of DEGs in each of the four states. **Table S6.** Gene ontology (GO) terms enriched in DEGs identified in each pairwise comparison of states. **Table S7.** KEGG and Reactome pathways enriched in DEGs identified in each pairwise comparison of states. **Table S8.** Summary of KEGG and Reactome pathways and GO terms enriched in DEGs in each WGCNA module. **Table S9.** Details of DEGs included in PPI network of module TP-M9 associated with Torpor. **Table S10.** Details of DEGs included in PPI network of TP-M10 module associated with Torpor. **Table S11.** Details of DEGs included in PPI network of module AS-M1 associated with Arousal. **Table S12.** DEGs upregulated in winter torpor relative to summer active that were shared by the three tissues (intestine, liver and brain) in *Rhinolophus ferrumequinum*.

## Data Availability

The raw sequencing reads were deposited in the NCBI Sequence Read Archive (SRA) under Bioproject PRJNA638701, SRR12006225-SRR12006254. Assembly of the reference transcriptome is available in Dryad: 10.5061/dryad.3n5tb2rd8. The datasets used and/or analysed during the current study are available from the corresponding author on reasonable request. All data generated or analysed during this study are included in this published article and its supplementary information files.

## Abbreviations

BUSCO: Benchmarking Universal Single-Copy Orthologs
DEGs: Differentially expressed genes
ER-phagy: Endoplasmic reticulum-phagy
FDR: False discovery rate
GO: Gene Ontology
Log_2_FC: Log_2_ transformed fold change
ORFs: Open reading frames
MM: Module membership value
nr: Non-redundant protein database
PCA: Principal component analysis
PPI: Protein-protein interaction
RNA-Seq: RNA sequencing
SRA: Sequence read archive database
Tb: Rectal temperature
TOM: Topological overlap matrix
WGCNA: Weighted gene co-expression network analysis
Genes or proteins, ALB: Albumin
CCK: Cholecystokinin
HPX: Hemopexin
HP: Haptoglobin
CSF1: Macrophage colony-stimulating factor
PDH: Phosphorylate pyruvate dehydrogenase
TXNIP: Thioredoxin-interacting protein
GPR17: G protein-coupled receptor 17
