# Supplementary figures and images for: Gut transcriptomic changes during hibernation in the greater horseshoe bat (Rhinolophus ferrumequinum)
Source: Front Zool. 2020 Jul 17;17:21. doi: 10.1186/s12983-020-00366-w (PMC7366455; doi:10.1186/s12983-020-00366-w)

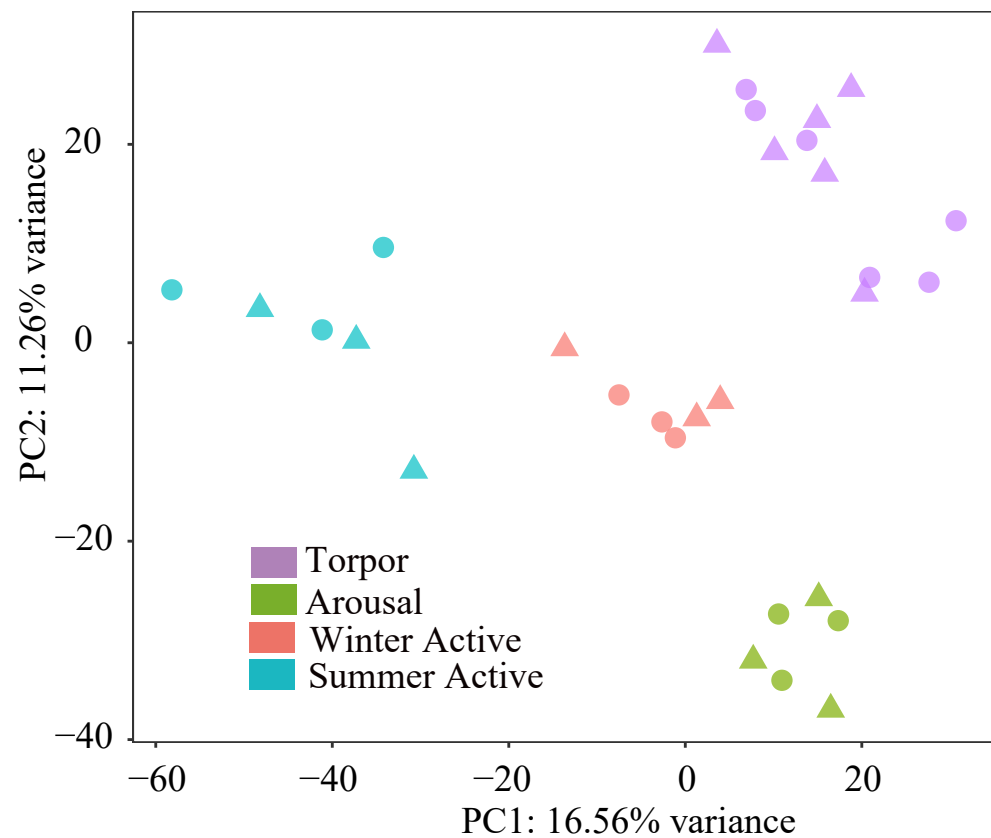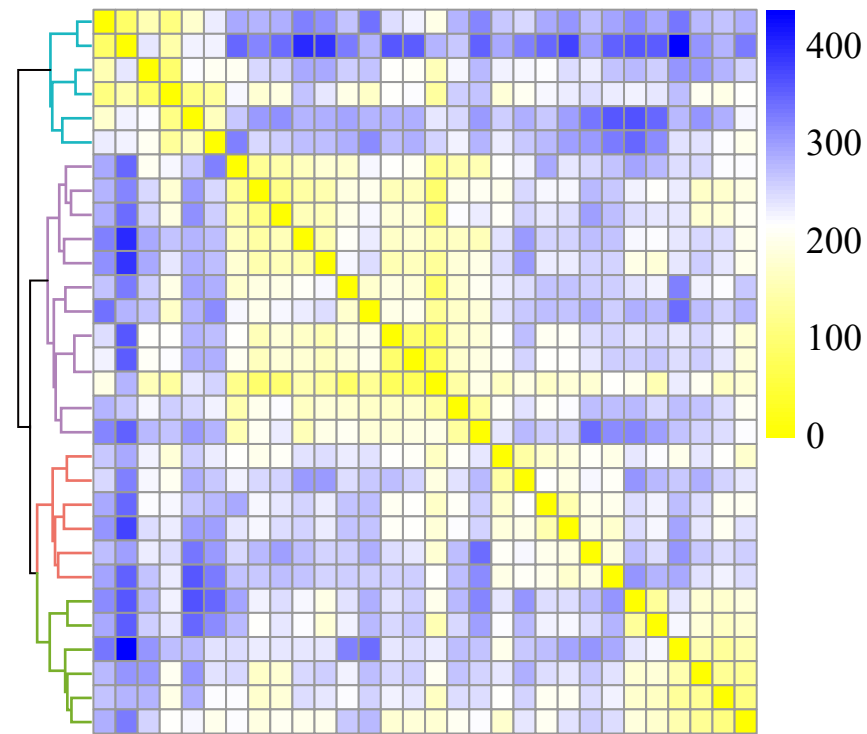

Supplement: Supplementary file 1 — Additional file 1: Figure S1. Principal component analysis (PCA) and clustering of the 30 samples from 15 individuals based on expression data of 14,009 genes showing gene expression distance of all samples. [file 12983_2020_366_MOESM1_ESM.pdf]

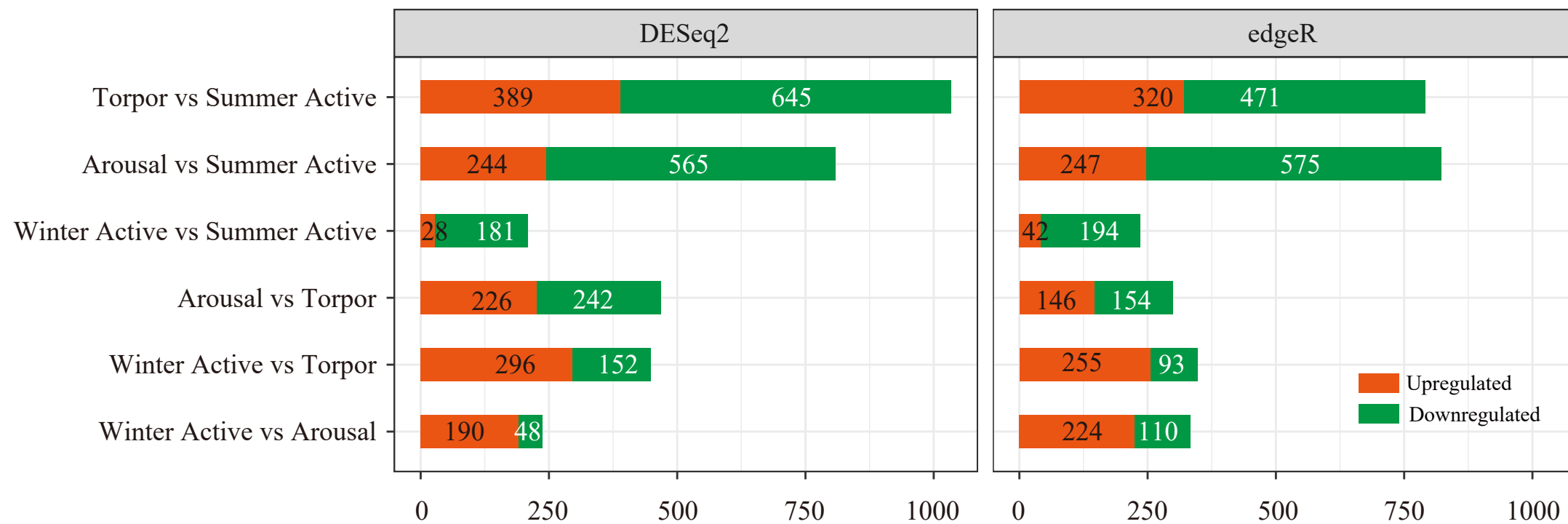

Supplement: Supplementary file 2 — Additional file 2: Figure S2. Number of differentially expressed genes (DEGs) identified in each pairwise comparison of the four states by DESeq2 and edgeR, respectively. [file 12983_2020_366_MOESM2_ESM.pdf]

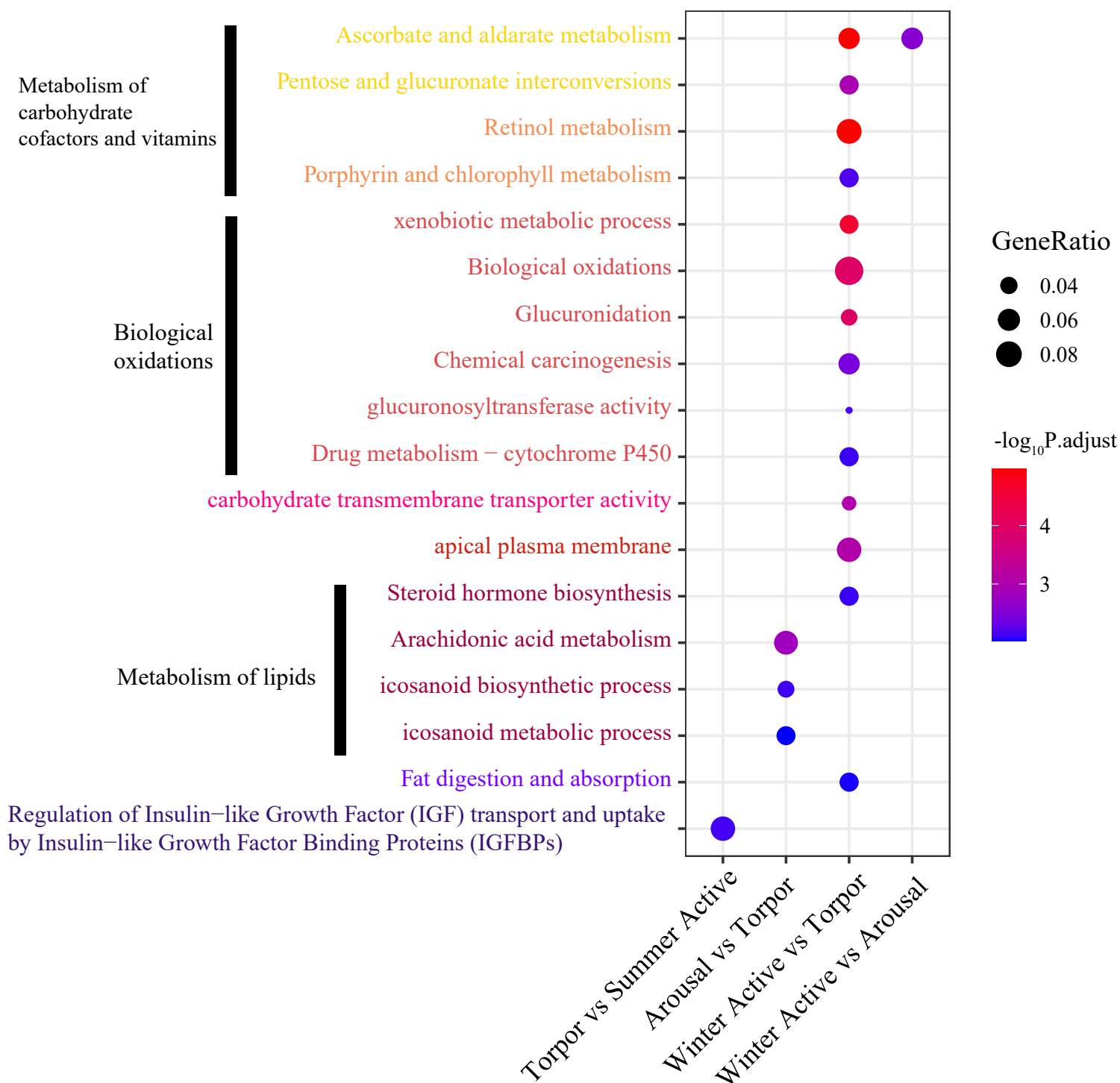

Supplement: Supplementary file 3 — Additional file 3: Figure S3. Results of GO terms and/or KEGG and Reactome pathway enrichment analysis on upregulated DEGs identified in four comparisons. Description of terms or pathways with same color indicates similar function. [file 12983_2020_366_MOESM3_ESM.pdf]

Immune function

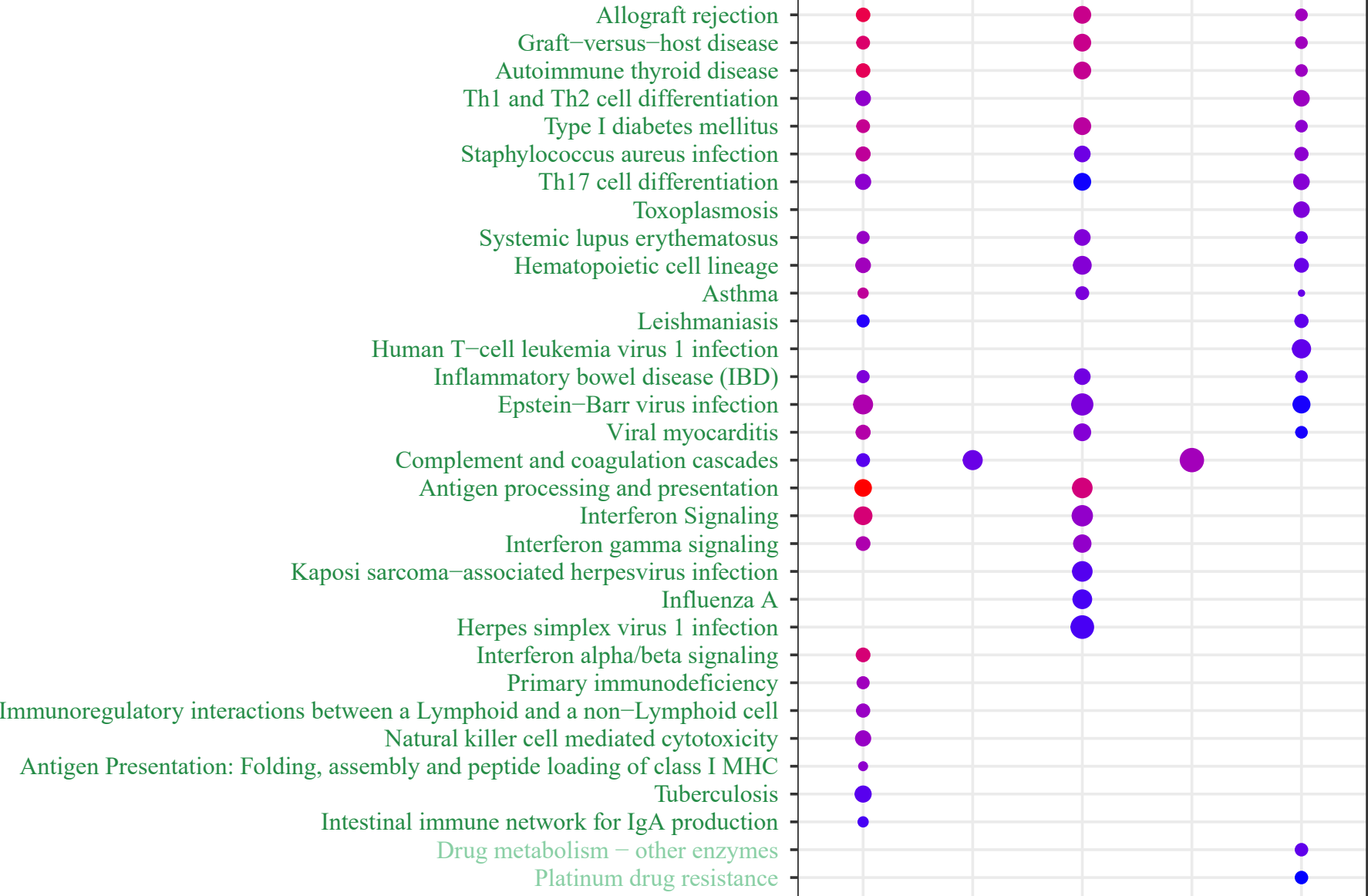

Hemostasis

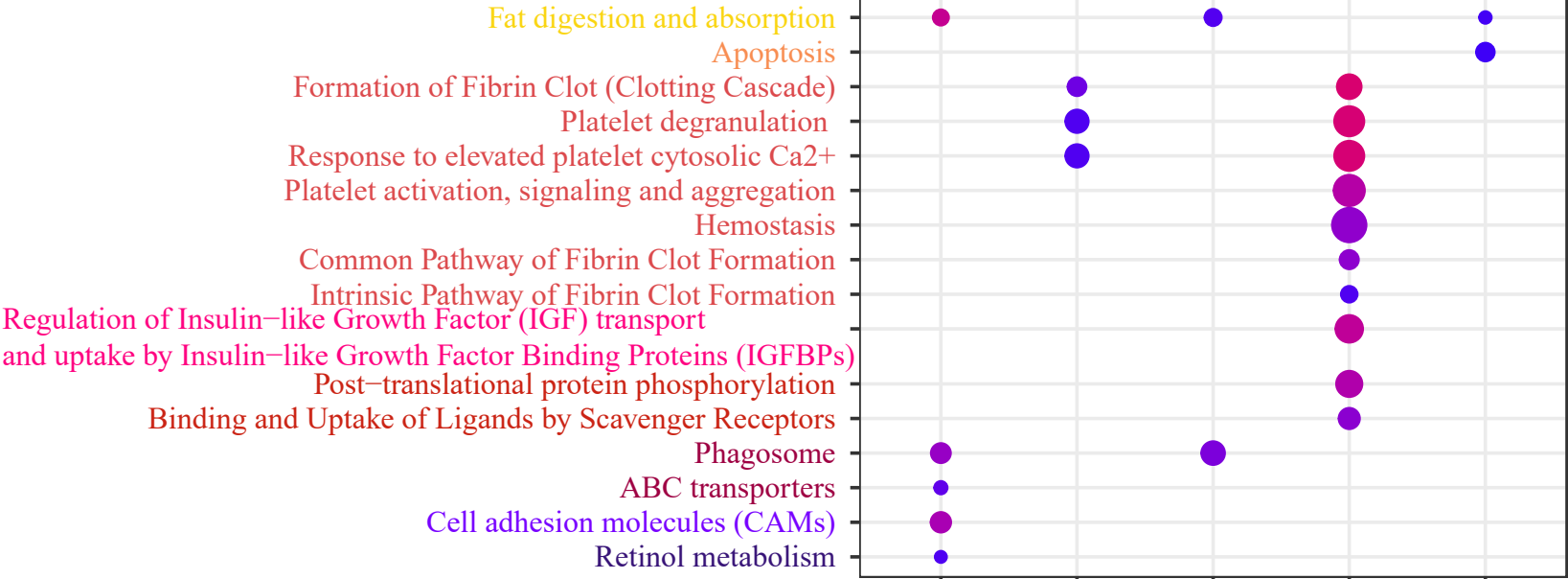

Metabolism of proteins

Supplement: Supplementary file 4 — Additional file 4: Figure S4.Results of KEGG and Reactome pathway enrichment analysis on downregulated DEGs identified in five comparisons. Description of terms or pathways with same color indicates similar function. [file 12983_2020_366_MOESM4_ESM.pdf]

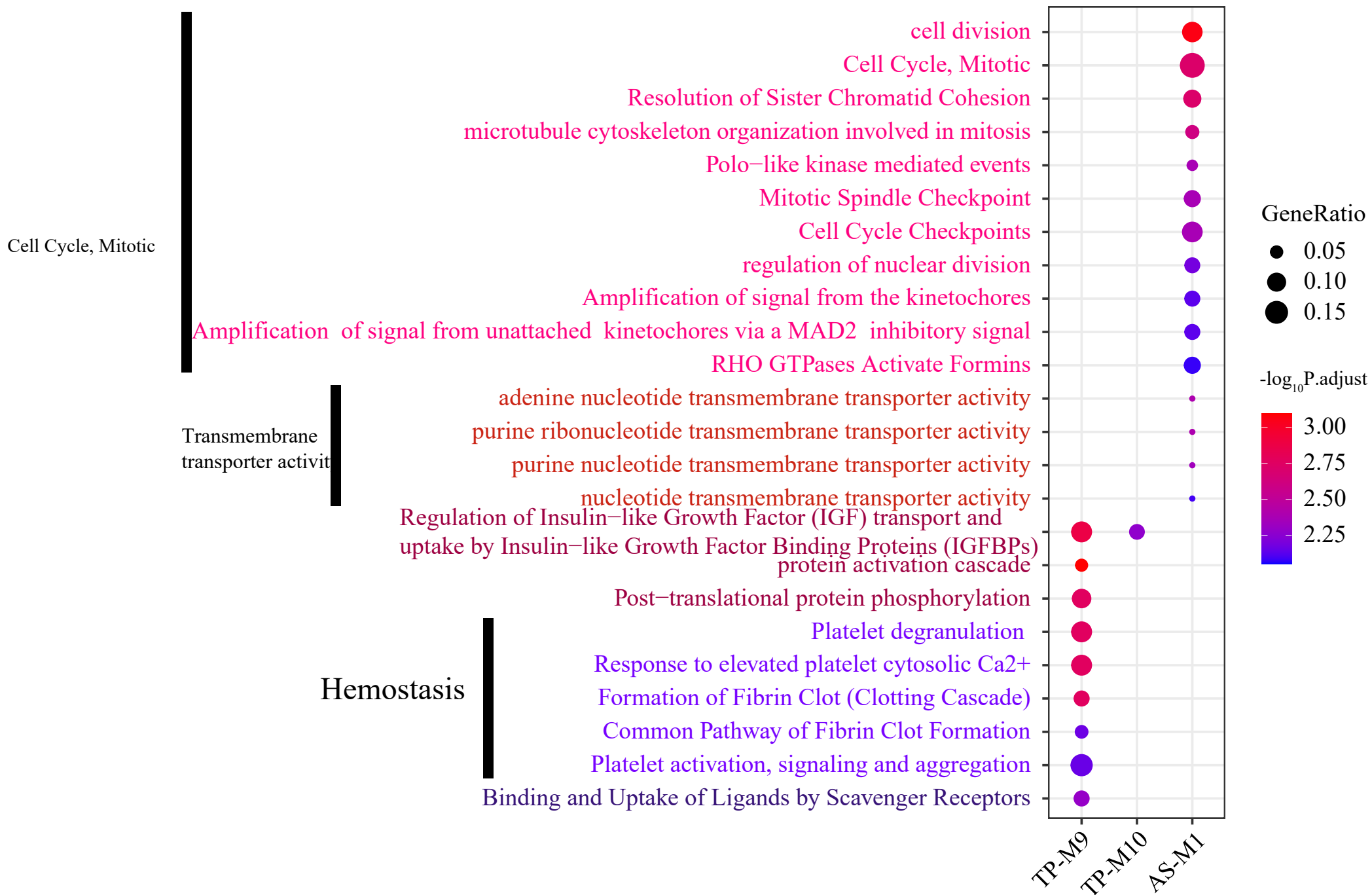

Supplement: Supplementary file 5 — Additional file 5: Figure S5.Results of GO terms and/or KEGG and Reactome pathway functional enrichment analysis on genes in TP-M9, TP-M10 and AS-M1 modules identified in WGCNA. Description of terms or pathways with same color indicates similar function. [file 12983_2020_366_MOESM5_ESM.pdf]
